# HLA, infections and inflammation in early stages of atherosclerosis in children with type 1 diabetes

**DOI:** 10.1007/s00592-017-1063-1

**Published:** 2017-10-24

**Authors:** Michal Odermarsky, Erkki Pesonen, Timo Sorsa, Åke Lernmark, Pirkko J. Pussinen, Petru Liuba

**Affiliations:** 1 Department of Paediatric Cardiology, Paediatric Heart Center, Lund University and Skåne University Hospital, 22185 Lund, Sweden; 2 Division of Periodontology, Department of Dental Medicine, Karolinska Institutet, Huddinge, Sweden; 3 Department of Clinical Sciences, Lund University, Skåne University Hospital, Malmö, Sweden; 4 Department of Oral and Maxillofacial Diseases, University of Helsinki, Helsinki, Finland

**Keywords:** Children, Intima-media thickness, Type 1 diabetes, Infections, Matrix metalloproteinase-8

## Abstract

**Aims:**

This prospective study focuses on risk factors for arterial damage in children with type 1 diabetes (T1D).

**Methods:**

Eighty children and adolescents with T1D were investigated twice, approximately 2 years apart, for carotid artery intima-media thickness (cIMT) and compliance (CAC), flow-mediated dilatation (FMD) of the brachial artery, and plasma levels of matrix metalloproteinase (MMP)-8. All subjects were genotyped for HLA. The number of respiratory tract infections (RTI) during the past year was obtained by a questionnaire in 56 patients.

**Results:**

cIMT progression, defined as percentage (%) change of cIMT from baseline, correlated inversely with the % changes of both CAC (*p* = 0.04, *r* = − 0.3; *n* = 62) and FMD (*p* = 0.03, *r* = − 0.3; *n* = 47). In multivariate analysis, RTI frequency correlated significantly with cIMT progression irrespective of age, diabetes duration, BMI, and HbA1c (*p* = 0.03, *r* = 0.3). When patients were divided in relation to RTI, the association of DQ2/8 with cIMT progression remained significant in patients with over three infections/year (*p* = 0.04, *r* = 0.3). During follow-up, the group of DQ2/8 patients with hsCRP > 1 mg/l showed significantly higher levels of plasma MMP-8 than the non-DQ2/8 group.

**Conclusions:**

The diabetes-risk genotype DQ2/8 and systemic inflammation contribute to pro-atherosclerotic vascular changes in children and adolescents with T1D.

## Introduction

Increased infection recurrence appears to be independent predictor of the risk of developing T1D [1] and impaired carotid artery elasticity [2], an index of early atherosclerosis [3]. Endothelial cell damage and inflammation are central in atherogenesis. Inflammation is involved not only in the initiation and progression of atherosclerosis, but also in the occurrence of its complications such as myocardial infarction and stroke [4]. Both bacterial and viral infections correlate to preatherosclerotic changes in the arteries and coronary disease [5]. Experimental animal studies have more consistently provided evidence for deleterious effects of various infectious pathogens on arterial structure [6–8]. These effects seem to be more prominent in vessels from younger animals, and appear to correlate with the number of inoculations [9]. Nonetheless, negative studies have been reported as well [10].

Atherosclerosis develops faster in children with T1D [11]. Several genes within the HLA region encoding antigen-presenting molecules with role in the autoimmune cascade in T1D have been shown to associate with damage to vascular endothelium in vitro [12]. Experimental studies have shown that, in an inflammatory milieu, HLA may be quickly translocated to endothelial cell surface, thereby contributing via antigen presentation to intravascular (in arteries) or extravasal (in microcirculation) accumulation of T lymphocytes [12, 13].

The genetic HLA-related susceptibility is recognized in more than 80% of young patients with T1D [14]. We have earlier reported in a cross-sectional study of children and young adults with T1D that diabetes duration, body mass index, and glycosylated haemoglobin were strongly associated with brachial artery and cutaneous microvascular endothelial dysfunction in patients with HLA-DQ 2/8 but not in those without this genotype [15, 16]. The risk of endothelial damage seemed to increase further in patients with inflammatory activity, suggesting a possible interplay between systemic inflammation and HLA in vascular injury.

The HLA-mediated presentation of autoantigens by vascular endothelial cells, a key-event in the transmigration of auto reactive T-cells and diabetes development, seems to occur systemically [13]. This could perhaps explain previous findings of elevated levels of endothelial cell activation markers in children with genetic risk for T1D before the onset of the disease [17].

Serum and plasma matrix metalloproteinase-8 (MMP-8) levels have been demonstrated to reflect disease severity and plaque instability in cardiovascular diseases [18]. MMP-8 concentrations are increased during acute coronary syndrome [19] and in young individuals with subclinical atherosclerosis [20].

In the present prospective study, we investigated whether DQ2/8, acute infections and plasma MMP-8 concentrations in children and young adults with T1D associate with adverse changes in arterial function and intima-media thickness.

## Materials and methods

### Study population

Eighty children and adolescents (37 male and 43 female) with age between 8 and 20 (mean age 15) years with T1D duration for at least 6 months were recruited from the paediatric outpatient diabetes clinic at the Lund University Hospital. All patients were treated with daily insulin injections. Exclusion criteria were: family history for other major cardiovascular risk factors (primary hypercholesterolemia, hypertension, premature coronary and cerebrovascular disease), active smoking, systemic hypertension, asthma and allergy. None of the patients were on corticosteroids or other type of immunosuppressive medication. All patients were followed for 1.40 ± 0.02 years (range 1.1–2.0 years).

Body weight, height, and arterial blood pressure (systolic and diastolic) were measured at both visits. Data on demographic information were collected via questionnaire. Data on diabetes duration were obtained from the registry of the outpatient diabetes clinic.

Detailed infection history including type and number of acute infections during the past year, duration and severity of symptoms, medication if any, and time between the latest infection and the ultrasound visits was also determined by questionnaire. Acute infection was defined by clinical symptoms. Patients were grouped in relation to the number of infections during the past year as follows: group 1 = 0 to 1 infection (low frequency), group 2 = 2 to 3 infections (moderate frequency), and group 3 = ≥ 3 infections (high frequency).

Patients were also grouped based on the hsCRP levels as follows: ≤ 1 and > 1.0 mg/l.

### Biochemical analyses

All blood samples were taken in connection with the ultrasound examination, and stored at − 80 °C until final analysis. High-density lipoprotein (HDL-C), low-density lipoprotein (LDL-C) and total cholesterol (TC) were analysed from lithium heparin plasma by enzymatic method (Roche/Hitachi 912, Roche Diagnostics, Mannheim, Germany). Plasma high-sensitivity C-reactive protein (hsCRP) was measured by enzyme-linked immunoassay using polyclonal antibodies (DACO Diagnostics, Glostrup, Denmark).

HLA genotypes were determined in dried spots of peripheral blood by polymerase chain reaction followed by DELFIA^®^ hybridization assay [21]. Briefly, DNA in the blood was amplified, and presence of particular alleles was determined by a hybridization reaction using allele-specific, short oligonucleotides labelled with lanthanide chelates.

Plasma MMP-8 concentration was determined by a time-resolved immunofluorometric assay (IFMA). The monoclonal MMP-8-specific antibodies 8708 and 8706 (Medix Biochemica, Kauniainen, Finland) were used as capturing and detection antibody, respectively. The detection antibody was labeled using europium chelate [20]. The assay buffer was 20 mM Tris–HCl, pH 7.5, containing 0.5 M NaCl, 5 mM CaCl_2_, 50 μM ZnCl_2_, 0.5% BSA, 0.05% sodium azide and 20 mg/l diethylenetriaminepentaacetic acid. Samples were diluted 1:5 (vol:vol) in the assay buffer and incubated for 1 h, followed by incubation for 1 h with the detection antibody. Enhancement solution was added and fluorescence was measured after 5 min using a 1234 Delfia Research Fluorometer (Wallac, Turku, Finland). The inter-assay coefficient of variation was 7.3% (*n* = 28). The detection limit for the assay was 0.08 ng/ml.

### Carotid artery intima-media thickness and compliance

A high-resolution ultrasound system (Acuson Sequoia C512, Siemens AG, Germany) equipped with a 15 MHz probe was used [31]. Longitudinal scans in bi-dimensional mode of the 1-cm-long distal end of the left common carotid artery were imaged so that the lumen-intima and media-adventitia interfaces were distinguishable. All images corresponded to the R-wave on electrocardiogram (ECG). Four scans obtained from each individual were recorded on videotape for offline analysis of carotid intima-media thickness (cIMT). Two sonographers unaware of the infection and genetic status carried out the ultrasound examination. The mean cIMT was measured from each scan manually. cIMT obtained from all scans from the same subject were averaged and the resulted mean cIMT was used for statistical analyses. The percent change of cIMT (% cIMT) was calculated. Carotid artery compliance (CAC) was calculated according to the following formulas: CAC = ([*D*_s_ − *D*_d_]/*D*_d_)/(*P*_s_ − *P*_d_), where *D*_s_ is systolic diameter, *D*_d_ is diastolic diameter, *P*_s_ is systolic blood pressure, and *P*_d_ is diastolic blood pressure. CAC reflects the ability of arteries to expand in response to the pulse pressure caused by cardiac contraction and relaxation. In a prior reproducibility study on 10 randomly selected patients, the coefficient of inter-observer variability was 6.2%.

### Flow-mediated dilatation

Longitudinal scans of the brachial artery (non-dominant arm) were imaged several centimetres above the antecubital fossa via a 15-MHz linear ultrasound transducer of an Acuson Sequoia^™^ C512 (Siemens AG, Germany) [32]. The ultrasound beam frequency was set at 8 MHz. Once the image was obtained, the transducer was positioned throughout the ultrasound study with aid of a transducer arm. ECG-gated end-diastolic scans of the artery were recorded at baseline, and a pressure cuff tourniquet placed around the forearm was thereafter inflated to 200 mmHg (minimum 50 mmHg over the systolic blood pressure) for 5 min. A new series of frames were taken for 15 s before and 120 s after cuff deflation. Following a 10-min recovery period, additional frames were taken before and over a 4-min period after sublingual administration of 400 µg glycerol trinitrate (GTN spray). The latter was used as endothelium-independent vasodilator. Flow-mediated and GTN-induced brachial artery dilatations were expressed as maximum percent dilatation following cuff deflation and GTN administration, respectively.

### Statistical analysis

Differences in the studied variables were assessed by ANOVA, and adjusted for covariates by ANCOVA. Eventual correlations between the hypothesised predictor variables and the dependent variable were assessed by logistic regression. When significant, multiple regression models were used to identify independent factors affecting the vascular indexes. hsCRP was logarithmically transformed due to its skewed distribution. All other numeric variables showed a Gaussian distribution. “cIMT progression” was defined as % increase in cIMT at 18 months from the baseline. Similarly, changes in FMD and CAC over the follow-up period were expressed as % change at 18 months from the corresponding baseline values.

All data are expressed as mean ± SD unless otherwise specified. Statistical significance was accepted when p was less or equal to 0.05. All statistical analyses were performed with StatView for Windows (version 5.0, SAS Institute, USA).

## Results

**Fig. 1:**
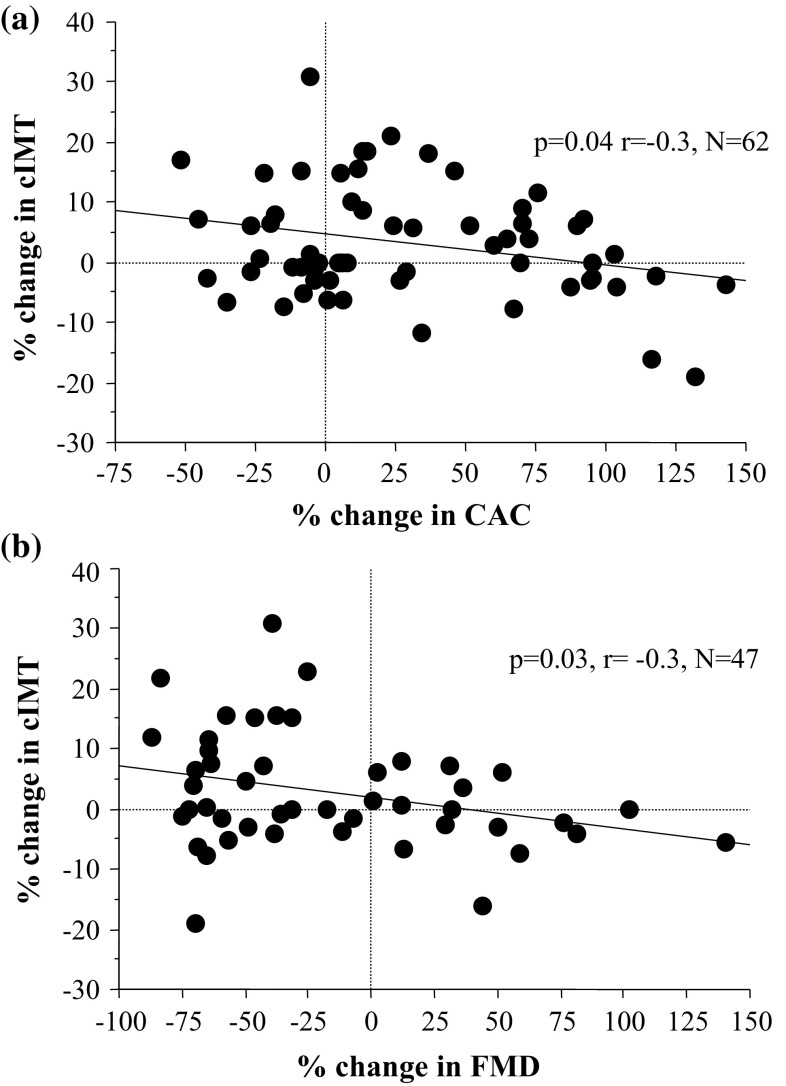
Scatterplot of % change in cIMT and CAC (panel **a**) and FMD (panel **b**)

The patients’ main characteristics according to the DQ2/8 genotype are summarised in Table 1. As shown in Table 1, there were no significant differences in the demographic, diabetes, and inflammatory variables. There was no correlation between diabetes duration and number of RTI (*p* = 0.23, *r* = 0.13). Baseline diabetes duration (*p* = 0.04, *r* = 0.23) and BMI (*p* = 0.06, *r* = 0.20), but not HbA1c, had a weak association with the final cIMT, but neither of these showed a significant correlation with % cIMT (*p* > 0.2). % cIMT correlated inversely with the % changes in CAC (*p* = 0.04, *r* = − 0.3, *n* = 62; Fig. 1a) and FMD (*p* = 0.03, *r* = − 0.3, *n* = 47; Fig. 1b).

**Table 1:** Patients’ main characteristics in relation to HLA-DQ2/8

	DQ2/8		Non-DQ2/8		p* (For baseline)
	Baseline	Follow-up	Baseline	Follow-up	
Number of patients	32	32	45	45	–
Age (years)	14.7 ± 3.8	15.7 ± 3.8	15.2 ± 3.0	16.2 ± 3.0	0.47
BMI	21.1 ± 3.8	22.1 ± 4.4	21.0 ± 3.1	21.6 ± 3.1	0.90
Diabetes duration (years)	6.5 ± 4.3	7.4 ± 4.3	7.6 ± 4.2	8.7 ± 4.1	0.27
HbA_1c_ (%)	7.0 ± 1.7	7.6 ± 1.6	7.0 ± 1.1	7.3 ± 1.3	0.95
HbA_1c_ (mmol/mol)	53 ± 19	59 ± 18	53 ± 12	56 ± 14	0.95
hsCRP (mg/l)	2.5 ± 4.1	1.4 ± 1.9	1.6 ± 3.1	1.9 ± 2.6	0.20
MMP-8 (ng/ml)	NA	27.2 ± 25.1	NA	30.1 ± 57.5	NA
LDL-C (mmol/l)	2.5 ± 1.0	2.5 ± 0.9	2.1 ± 0.7	2.3 ± 1.2	0.06
HDL-C (mmol/l)	1.6 ± 0.4	1.3 ± 0.3	1.6 ± 0.4	1.3 ± 0.5	0.73
LDL/HDL	1.7 ± 0.8	2.0 ± 0.9	1.4 ± 1.1	1.9 ± 0.9	0.27
Total Chol (mmol/l)	4.5 ± 1.1	4.2 ± 1.1	4.1 ± 0.8	3.7 ± 1.4	0.11
cIMT (mm)	0.040 ± 0.006	0.042 ± 0.005	0.040 ± 0.004	0.041 ± 0.004	0.67
cIMT (% change)	NA	7.4 ± 12.5	NA	1.9 ± 7.7	0.02
CAC (%/10 mmHg)	2.8 ± 1.7	3.8 ± 1.2	2.8 ± 1.0	3.9 ± 1.8	0.91
CAC (% change)	NA	33.6 ± 48.7	NA	26.2 ± 48.7	0.57
FMD (%)	8.1 ± 6.8	6.2 ± 2.0	9.9 ± 7.2	7.0 ± 3.0	0.30
FMD (% change)	NA	− 23.4 ± 47.1	NA	− 17.1 ± 53.1	0.69

Data are mean ± SD

*NA* not applicable

* For lipids and CRP, *p* value was calculated after adjustment for age, diabetes duration, BMI, and HbA1c

### HLA-DQ 2/8 and vascular changes

**Fig. 2:**
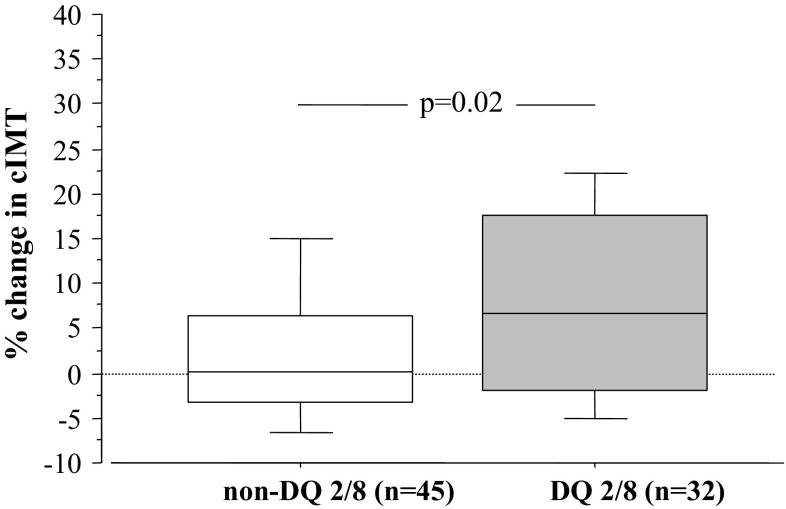
Box plot illustrating the difference in % cIMT between the DQ2/8 and non-DQ2/8 groups. The box plot displays the 25th percentile, median, and 75th percentile, as well as the 10th and 90th percentiles as horizontal lines outside the box

**Fig. 3:**
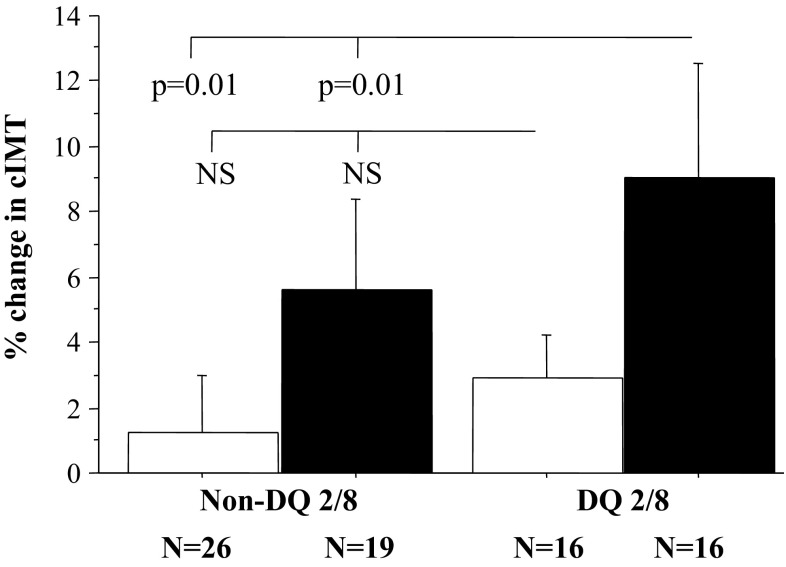
Bars (mean ± SD) illustrating the difference in % cIMT between the DQ2/8 and non-DQ2/8 groups divided in relation to gender (white bars = female; black bars = male). *NS* not significant

% cIMT was increased in the DQ2/8 group compared to the non-DQ2/8 group (7.4 ± 12.5 vs. 1.9 ± 7.7, respectively, *p* = 0.02, Fig. 2), whereas % FMD and % CAC were comparable in the groups (*p* > 0.5 for each). The relationship between % cIMT and DQ2/8 remained significant after simultaneous adjustment for age, BMI, diabetes duration, HbA1c, and LDL-to-HDL ratio (*p* = 0.03 by ANCOVA). Although gender alone did not influence % cIMT (male vs. female: *p* = 0.3), % cIMT was most increased in male DQ2/8 patients (Fig. 3; *p* = 0.01 vs. female non-DQ2/8 patients; *p* = 0.04 after adjustment for age, BMI, diabetes duration, HbA1c, and LDL-to-HDL ratio).

**Fig. 4:**
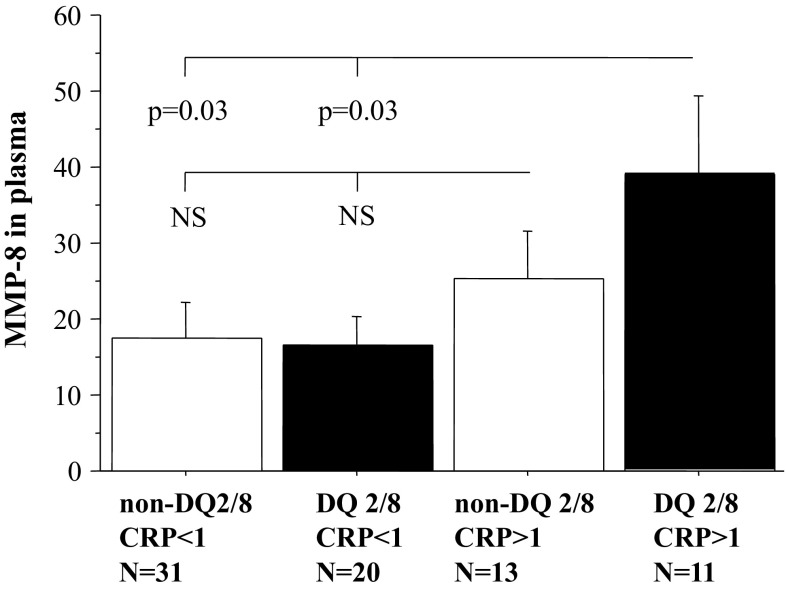
Bars (mean ± SD) illustrating the difference in plasma MMP-8 between the DQ2/8 and the non-DQ2/8 groups divided in relation to CRP (cut-off 1 mg/L), *NS* not significant

There was no significant difference in MMP-8 concentrations between DQ2/8 groups (*p* = 0.4). MMP-8 levels were significantly higher in those patients with hsCRP ≥ 1 compared to those with hsCRP ≤ 1at the follow-up (*p* = 0.045). However, only patients positive for DQ2/8 with hsCRP > 1 mg/L at follow-up had significantly higher levels of plasma MMP-8 than the non-DQ2/8 patients (Fig. 4). With exception of baseline BMI (*p* = 0.03, *r* = 0.3) and HbA1c (*p* = 0.04, *r* = 0.2), which were modestly associated with final MMP-8, no other variable showed correlation with it.

### Infection frequency and % cIMT

**Fig. 5:**
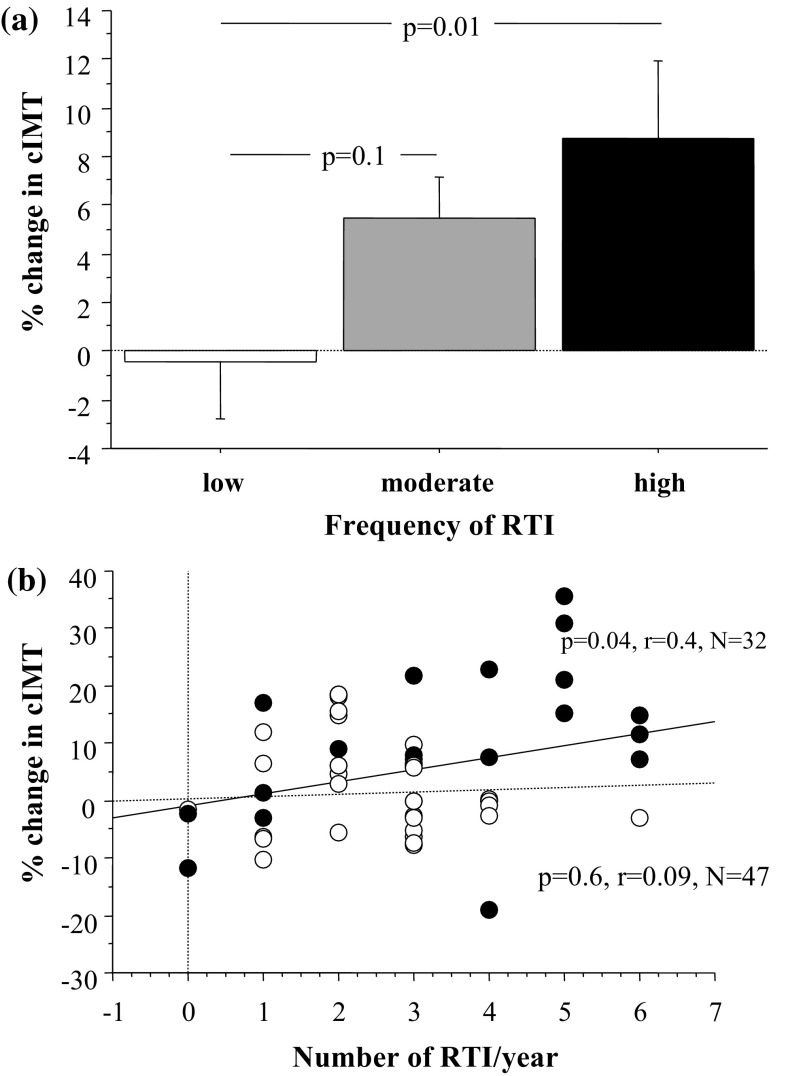
**a** Differences in % change in cIMT between patients grouped in relation to number of respiratory tract infections (RTI) during the preceding year. **b** Correlation between RTI frequency/year and % change in cIMT in patients with (black circles) and without HLA-DQ2/8 (white circles)

In a multivariate model adjusted for age, BMI, diabetes duration, HbA1c, and the number of RTI during the year prior to the baseline ultrasound, infection frequency showed a significant relationship to % cIMT (Table 2). % cIMT was most increased in patients with > 3 RTI/year (Fig. 5a). When patients were divided on the basis of the genotype, the correlation between infection frequency and % cIMT remained significant only in DQ2/8 patients (Fig. 5b).

**Table 2:** Multivariable regression analysis of % change in cIMT

	Coefficient	Std error	Std coefficient	*t*-value	*p* value
Intercept	1.852	12.408	1.852	0.149	0.8820
Age (years)	− 0.147	0.620	− 0.046	− 0.0238	0.8130
BMI (kg/m^2^)	0.045	0.0565	0.014	0.080	0.9171
HbA1c (%)	− 0.141	1.351	− 0.017	− 0.105	0.9171
Diabetes duration (years)	− 0.095	0.435	− 0.039	− 0.219	0.8273
Infection frequency (*n*)	2.169	0.962	0.325	2.254	0.0285

## Discussion

There is solid evidence for association of T1D with premature carotid atherosclerosis in childhood [11]. In the present study of diabetic children and adolescents with and without HLA DQ2/8, we found suggestive evidence of accelerated structural changes (expressed as increase of % IMT) in those positive for this genotype. Carotid artery intima-media thickness increased most significantly in DQ2/8 patients that experienced three or more respiratory tract infections per year. Of note, the % change of cIMT was inversely related to the % change in both CAC and FMD. Neither CAC nor FMD did change significantly over the study period in relation DQ2/8. Similar to our study, Halcox et al. [22] in 2009 found decrease of FMD with averaged annual progression of cIMT.

Matrix metalloproteinases (MMPs) are structurally related but genetically distinct enzymes that can degrade almost all extracellular matrix components. Especially MMP-8 or collagenase-2 can also process various non-matrix bioactive substrates. Due to such decisive substrate cleavage(s) MMP-8 can mediate surrogate destructive pro-inflammatory immune reactions [23–25]. Many inflammatory cells secrete MMPs, which remodel the matrix facilitating thus the leukocyte traffic through tissues [26]. Among DQ2/8 patients, CRP levels > 1 mg/l was associated with elevated MMP-8, a biomarker of both systemic inflammatory and intravascular inflammation. In adults, high serum MMP-8 is an independent risk factor for incident cardiovascular events [20].

Functional disturbances in the vasculature such as endothelial vasomotor dysfunction and decreased arterial elasticity are important signs of early vascular disease, and associate with arterial intima thickening [27]. In a prior cross-sectional study from our centre, higher frequency of respiratory tract infections in T1D was linked to impaired carotid artery elasticity especially in those exposed to passive tobacco smoking [2]. Infection is a powerful trigger of inflammation in the body, being earlier associated to arterial endothelial cell damage and intima-media thickening in mice, piglets and children [28–30].

Even children without conventional risk factors for atherosclerosis appear to be prone to carotid artery thickening already 3 months after an acute infectious illness [31]. Acute infections initiate endothelial cell dysfunction in children, and the dysfunction is worse in diabetic children than in healthy children [31]. In the present study carotid artery intima-media thickness increased most significantly in DQ2/8 patients that experienced three or more respiratory tract infections per year. In adults, acute respiratory infections increased the risk of acute myocardial infarction [32]. Symptoms of acute infections associated with increased viral and bacterial titres and increased levels of inflammatory markers precede acute coronary events [33].

Intuitively, given the upregulatory effects exerted by systemic inflammation on both vascular adhesion molecules and HLA molecules, their putative detrimental influences on the vascular wall would become stronger in an inflammatory environment. In patients with rheumatoid arthritis (RA), another cardiovascular risk factor with important genetic susceptibility, the mortality from cardiovascular disease was most increased in patients with RA-risk HLA and inflammatory activity [34].

Future large-scale studies of young patients with T1D are needed to confirm these findings and, if proven, to provide additional mechanistic insights into the putative HLA-infection interplay on vascular wall. Eventual relationship between the observed structural changes and accelerated atherosclerosis needs to be investigated as well.

## Abbreviations

CAC: Carotid artery compliance
cIMT: Carotid artery intima-media thickness
FMD: Flow-mediated dilatation
HLA: Human leucocyte antigen
MMP-8: Matrix metalloproteinase-8
T1D: Type 1 diabetes
